# TLR7/8 ligands R848 and imiquimod induce differentiation of bone marrow cells from patients with myelodysplastic syndrome towards mature neutrophils

**DOI:** 10.1038/s41598-025-15859-z

**Published:** 2025-08-26

**Authors:** Eva Villamón, Paula Guerrero, María Luisa Gil, Iván Martín, Paula Amat, Daniel Gozalbo, Alberto Yáñez

**Affiliations:** 1 Servicio de Hematología, Hospital Clínico Universitario-INCLIVA, Valencia, Spain; 2 Instituto de Biotecnología y Biomedicina, BIOTECMED, and Departamento de Microbiología y Ecología, Universitat de València, Burjassot, Spain

**Keywords:** Myelodysplastic syndromes, Toll-like receptors, Bone marrow cells, Imiquimod, R848, Primary cultures, In vitro differentiation, Flow cytometry, t-SNE, Toll-like receptors, Myelodysplastic syndrome

## Abstract

Myelodysplastic syndromes (MDS) and acute myeloid leukaemia (AML) arise as a consequence of acquisition and progressive accumulation of genetic and epigenetic modifications by haematopoietic stem and progenitor cells (HSPC) which result in an impaired cell differentiation and the clonal expansion of myeloid progenitors leading to blast-cell accumulation in bone marrow (BM) and myelodysplasia. TLRs are expressed on HSPC and play a role in modulating haematopoiesis by instructing commitment to the myeloid lineage, and therefore may have potential therapeutic application. We have determined the in vitro effect of R848 (TLR7/TLR8 agonist) and Imiquimod (TLR7 agonist), on differentiation, apoptosis and cell viability in primary cultures of bone marrow samples from MDS (n = 6) and AML patients (n = 13). Differentiation was determined by a combined approach of conventional flow cytometry and t-SNE (t-distributed stochastic neighbour embedding) analysis based on the expression of cell markers (CD34, CD11b, CD13, CD117 and CD45). Cell viability and apoptosis were determined according to standard procedures. Statistical analyses were performed according to the two-tailed Student’s *t* test for dual comparison (treated versus control samples). All major cell populations of the differentiation path from blasts towards neutrophils were found. Treatment with R848 or with Imiquimod did not induce significant changes in cell differentiation in AML samples. However, both R848 and, to a lesser extent, Imiquimod were able to induce differentiation of bone marrow cells from MDS patients from myelocytes to mature neutrophils in five out of six samples. Results also showed absence of toxic effects of both ligands on cells from MDS patients, as both apoptosis and cell viability were not altered by treatments. As for the differentiation assays, the effect of both ligands on apoptosis and cell viability in primary cultures from AML patients was not significant. Treatment with TLR7/8 ligands can revert the blockade of myeloid differentiation in most MDS samples and increase the amount of neutrophils, and therefore could represent a potential alternative treatment for MDS patients.

## Introduction

Myelodysplastic syndromes (MDS) and acute myeloid leukaemia (AML) encompass a series of heterogeneous disorders which arise as a consequence of acquisition and progressive accumulation of genetic and epigenetic modifications by haematopoietic stem cells (HSC)^1–3^; these changes result in an impaired cell differentiation and the clonal expansion of myeloid progenitors in the bone marrow (BM) and peripheral blood leading to blast-cell accumulation in BM, myelodysplasia and insufficiencies in haematopoiesis, such as neutropenia^4–8^.

MDS is one of the most frequent haematological cancer, particularly in old adults, with a high mortality rate. According to their severity, MDS have been classified into several categories. High risk MDS are characterized by a high degree of dysplasia, at least 10% of blasts in BM, neutropenia, increased mortality rate and probability of evolving into AML. Neutropenia in patients with MDS increases the risk of serious infections, which are responsible for 38% of deaths^3,8–13^.

AML is the most frequent acute leukaemia in adults. The presence of specific genetic modifications determines AML classification in different risk-based categories. Approximately half of AML cases are de novo generated, while others may arise from other myeloid disorders such as MDS (29%). AML is an aggressive disorder, with patients prone to suffer haemorrhagic events and infections as a consequence of the low levels of erythrocytes, platelets and leucocytes. The prognosis is poor with and overall survival rate of 23% at five years, depending on the AML type^1,5,14,15^.

Currently the standard therapy for MDS and AML is based on chemotherapy treatments using cytotoxic molecules aimed at killing highly proliferating cells, despite the serious secondary effects and the risk of relapse. Failure of treatments is often due to drug resistance and dormancy of the leukemic stem cells with a diminished susceptibility to cytotoxic drugs^13,16^. Some patients can be treated with allogeneic transplant of stem cells, which may improve their survival, and the rapid advances in deciphering molecular pathogenesis pave the way towards precision medicine^17,18^. However, most patients do not overcome the disease in the long term^18–20^. Therefore, new strategies are needed to improve treatment and prognosis of patients with these disorders. During the last years, the differentiation therapy has emerged as an alternative possibility for developing less toxic and more effective treatments^21^. In this context, the use of all-trans retinoic acid (ATRA) as differentiation agent has been successfully used for treatment of most patients with acute promyelocytic leukaemia (up to 10% of AML), although it does not work for other AML nor for MDS, and so alternative differentiation therapies should be investigated^22^.

It is well stablished that members of the TLR family of pattern-recognition receptors, which recognize molecular signatures of microbial pathogens, play an essential role in mature myeloid cells by triggering innate immune responses and the subsequent development of adaptive immune responses. It has been demonstrated more recently that functional TLRs are also expressed on haematopoietic stem and progenitor cells (HSPC) and there is increasing evidence showing their role in modulating haematopoiesis during infection by instructing commitment of human bone marrow HSC to the myeloid lineage^23–26^. In fact, this could explain the spontaneous remission of some cases of AML associated to systemic bacterial infections^27,28^. Consequently, TLR-mediated signalling may represent an alternative approach for the development of novel therapeutic treatments for MDS and AML. Expression of functional TLRs have been reported in human AML and MDS cell lines^29–32^. Activation of TLR8 in primary mouse AML cell cultures promotes differentiation and inhibits growth^33^. We have previously reported that Imiquimod (a TLR7 agonist) is able to inhibit cell proliferation, while induces apoptosis, cell cycle alterations and upregulation of myeloid differentiation markers of human leukaemic cell lines. R848 (a TLR7/8 agonist) also causes growth inhibition in some cell lines, whereas a TLR8 agonist has no effect, suggesting that growth inhibition is dependent on TLR7-mediated signalling. Other TLR agonists were able to inhibit cell proliferation in particular cell lines, indicating that response to TLR challenge is dependent on the cell line^32^. Moreover, R848 induces dendritic-like cell differentiation of human and murine *DNMT3A*-mutant AML cells and extends the survival of *DNMT3A*-mutant AML-bearing mice^34^. Besides, Imiquimod induces tumour-selective apoptosis and arrest of cell cycle in some types of tumour cells, such as skin, prostate and endometrial cancer cells, and it has been already used as non-invasive topical treatment for viral warts and superficial basal carcinoma^35–38^. In addition, EAPB0503, an Imiquimod analogue, inhibits growth and induces apoptosis in chronic leukaemia cells^39^. The evolving role of R848 and Imiquimod in immunotherapy (monotherapy or in combined therapy) in preclinical studies and clinical trials of various cancer types has been recently reviewed^40^. Approval from the food and drug administration (FDA) was granted for the Imiquimod treatment of basal cell carcinoma in 2004, with high disease clearance rates observed in phase III clinical trials. Remarkably, clinical trials that aim to explore novel combinations and delivery strategies of Imiquimod and R848 are ongoing for the treatment of vulval intraepithelial neoplasia, high-grade cervical intraepithelial neoplasia, early-stage cutaneous T cell lymphoma and breast cancer cutaneous metastases. Moreover, clinical trials of combination therapies including for example TLR7/TLR8 agonists as adjuvants in vaccination are in progress for several cancer types^40^.

The anti-tumour activity of both, Imiquimod and R848, can be mediated upon direct TLR7 activation on tumour cells as well as through TLR7 activation in other immune cells that modulate cell-mediated immune responses to tumour cells^41–44^, and there is increasing evidence indicating that TLR7/8 agonists may directly stimulate antitumor responses in various murine cancers when combined with standard therapies^45–47^. All these observations point out that Imiquimod and R848 could be also considered as potential alternative therapy for MDS and AML^48^.

Based on this background, in the present work we study the effect of Imiquimod and R848 on the in vitro proliferation and differentiation of primary bone marrow cell cultures from patients with MDS and AML. We show that R848, and Imiquimod, in a lower extent, induce differentiation towards the myeloid lineage and increase the levels of mature neutrophils in cell cultures from MDS patients, whereas no significant effect was found in samples from AML patients. Our results support the notion that TLR7/TLR8 activation by their specific ligands can be considered as a potential therapeutic strategy for development of alternative MDS treatments.

## Materials and methods

### Patient samples

A total of 19 bone marrow samples from patients diagnosed with MDS (6 samples: patients 1–6) and AML (13 samples: patients 7–19) were used (Table 1). The patients were diagnosed according to the international consensus classification of myeloid neoplasms and acute leukaemia, based on the former classification by the World Health Organization, the American Society of Hematology and the European Association for Haematopathology^13,49–51^. The major diagnostic features of the patients are shown in Table 1. All samples were obtained prior to treatment at the Hospital Clínico Universitario of Valencia (Spain).

**Table 1:** Data from MDS and AML patients and main diagnosis features.

Patient	Sex/age	Diagnosis
1	Male/84	MDS with multilineage dysplasia. Low risk
2	Female/83	MDS with multilineage dysplasia. High risk
3	Male/83	MDS with ring sideroblasts
4	Male/85	MDS with multilineage dysplasia
5	Male/75	MDS with excess of type II blasts. High risk
6	Male/83	MDS with ring sideroblasts and multilineage dysplasia
7	Male/81	AML with dysplasia-related changes (M2)
8	Male/65	AML with dysplasia-related changes (M4)
9	Male/65	AML with dysplasia-related changes (M4)
10	Female/80	AML (M0)
11	Female/80	AML with dysplasia-related changes (M5)
12	Male/81	Acute myelomonocytic leukemia (M4)
13	Female/40	AML with mutated NPM1 (M4)
14	Male/43	AML with dysplasia-related changes (M1)
15	Female/57	AML with t(6/9) chromosomal translocation (M1)
16	Male/69	Acute myelomonocytic leukemia (M4)
17	Male/48	AML with maturation (M2)
18	Female/55	AML with dysplasia-related changes (M4)
19	Male/80	Acute myelomonocytic leukemia (M4)

All bone marrow samples were acquired from the biobank of Hospital Clínico Universitario of Valencia with written informed consent in accordance with the Declaration of Helsinki and the approval of the internal review of Bioethics and Medical Research of the Hospital Clínico Universitario of Valencia.

### Primary cell cultures and TLR ligands used

First, red blood cells were removed from bone marrow samples following incubation with lysis buffer (Becton Dickinson) according to standard procedures. Then, cells were pelleted by centrifugation (5 min at 500 g, at room temperature) and resuspended at a concentration of 10^6^ cells/mL in RPMI 1640 medium (Gibco) supplemented with 10% heat-inactivated fetal bovine serum, 1% of penicillin–streptomycin stock solution (Gibco) and 2 mM glutamine (complete medium). Cells were seeded in 96-well plates (100,000 cells in 100 µL per well) either in the absence (control cultures) or the presence of the following TLR ligands: Imiquimod (InvivoGen), a TLR7 agonist, at a concentration of 10 µg/mL, or R848 (InvivoGen), a TLR7/8 agonist, at a concentration of 25 µg/mL; these concentrations of both ligands have been previously used to study their effect on AML cell lines^32–34^. Cell cultures were incubated for 24 or 48 h at 37 °C in a humidified atmosphere containing 5% CO_2_.

### Measurement of cell viability

Cells were analysed for proliferation after 24 h of culture by a colorimetric method to quantify viable cells using the CellTiter 96 AQueous One Solution Cell Proliferation Assay (Promega) according to the manufacturer recommendations^32^. All samples were run in triplicate. The absorbance values at 450 nm were measured after 3 h of incubation at 37 °C and corrected by subtracting the values of background absorbance (cell-free culture media). Percentage of viable cells of each treated sample was calculated taking the values of their corresponding controls as 100%.

### Determination of apoptosis by flow cytometry

Apoptosis was determined by measuring annexin-V binding in cell samples by using the Annexin-V Apoptosis Detection kit (Santa Cruz Biotechnology) according to the manufacturer instructions^32^. Samples were analysed by flow cytometry in a FACS Canto II flow cytometer (Becton–Dickinson). Samples were analysed in duplicates and apoptosis is expressed as percentage of Annexin-V positive cells.

### Measurement of cell differentiation by immunophenotyping and flow cytometry

Cell differentiation was analysed after 24 h and 48 h of cell culture incubation by flow cytometry. Cell suspensions were labelled with various mouse anti-human antibodies, targeting surface markers expressed at various stages of differentiation to identify distinct cell subsets, according to standard procedures. The following antibodies were used in this study: allophycocyanin (APC)-labelled anti-CD34 (clone 8G12), fluorescein isothiocyanate (FITC)-labelled anti-CD11b (clone D12), phycoerythrin (PE)-labelled anti-CD13 (clone 138), PE-Cy7-labeled anti-CD117 (clone 104D2), APC-H7-labelled anti-CD45 (clone HI30), and PerCPCy5.5-labelled anti-HLADR (clone L243). All antibodies were from Becton Dickinson. Flow cytometry analyses were performed on an FACS Canto II cytometer (Becton Dickinson), and data were analysed with FlowJo 10 software.

### Flow cytometry analysis of cell differentiation visualization with t-distributed stochastic neighbour embedding

**Fig. 1:**
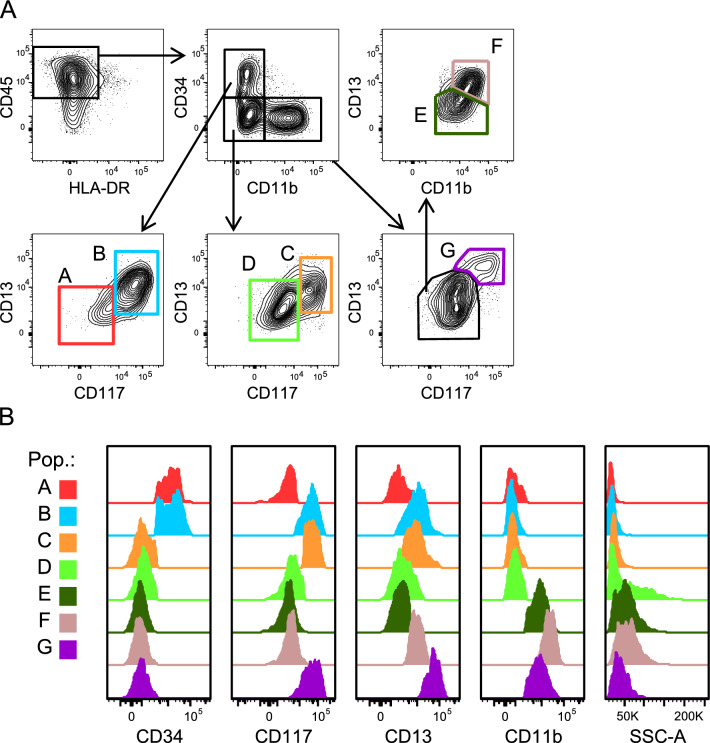
Flow cytometry analysis of the pooled non-treated and treated MDS and AML bone marrow cultures. (**A**) Manual gating strategy for selecting cell populations in the concatenated file. Colour boxes indicate the seven cell subsets identified in bone marrow cells lacking erythrocytes and lymphocytes (based on forward scatter and side scatter parameters, not shown). (**B**) Monoparametric histograms showing the fluorescence intensity of the cell markers used and side scatter (area) for each selected cell population. Population A (red), population B (blue), population C (orange), population D (light green), population E (dark green), population F (brown) and population G (purple).

In order to detect differentiated cell subsets related to the myeloid differentiation towards polymorphonuclear leucocytes (PMN), data from all 19 samples (both control and treated) were grouped into a single concatenated file. Analysis of the concatenated file enables the identification of cell subsets present across pooled patient samples (control non-treated and treated cell cultures). Sequential manual gating was performed on the concatenated file by selecting cell subsets in particular regions of a dot-plot bi-parametric graph according to the expression of cell surface markers (Fig. 1A). First, debris, dead cells, lymphocytes and doublets were excluded by forward scatter (FSC) and side scatter (SSC) parameters. Next, the cell population containing CD45^+^ (leucocytes) and HLA-DR^–^ cells (to exclude a minor population of mononuclear phagocytes) was selected. This cell population was analysed based on the expression of CD34 (a progenitor cell marker) and CD11b (a mature myeloid cell marker), resulting in the detection of three subsets: CD34^+^ CD11b^–^, CD34^–^ CD11b^–^ and CD34^–^ CD11b^+^, from low to high differentiation degree. These three cell subsets were further analysed based on the expression of CD117 (a progenitor cell marker) and CD13 (a marker of both progenitor and mature cells). This gating strategy allows the identification of seven cell subsets into the concatenated file (Fig. 1B).

**Fig. 2:**
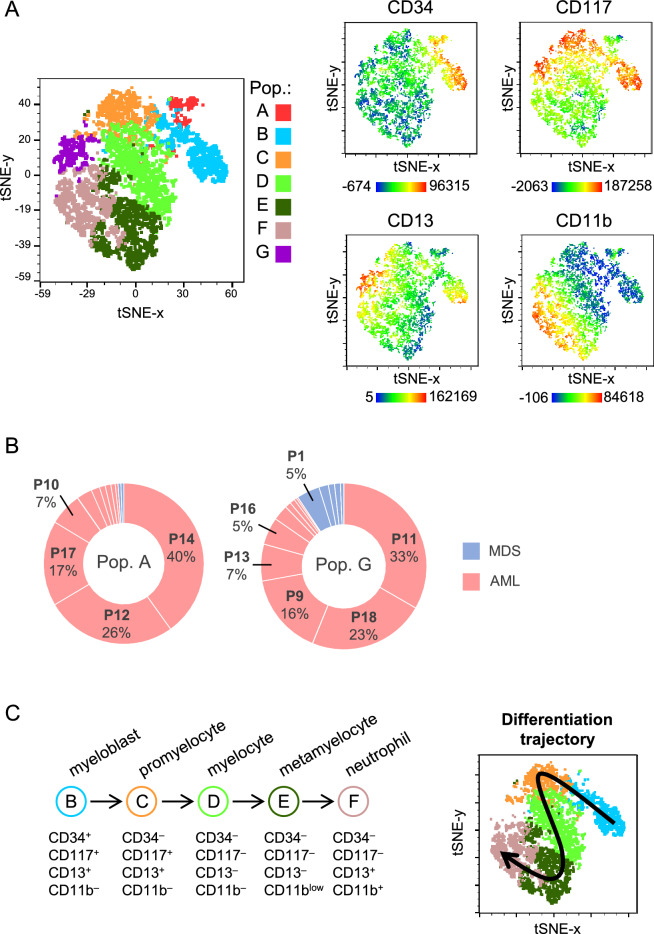
Results of the t-SNE analysis. (**A**) Cell populations identified in Fig. 1 are shown in a biparametric t-SNE-x and t-SNE-y graph following t-SNE manual gating of the concatenated file to create a single t-SNE map (left panel); heat map of the key phenotypic markers used for defining specific cell populations (right panel). (**B**) Quantitative graph showing populations A and G in MDS and AML samples. Single segments represent the percentage of the cell population in each patient, taken the pooled A or G cell populations as 100%, respectively; patients with higher percentages of cell populations are indicated. (**C**) Trajectory of the differentiation path towards mature neutrophils. The five identified cell populations (B-F) contain the cell subsets from myeloblast to neutrophils, according with the expression of their phenotypic markers (left); the arrow shows the trajectory of the differentiation path in the t-SNE graph (right).

The different cell clusters were shown by using the dimensionally reduction algorithm, t-distributed stochastic neighbour embedding SNE (t-SNE), to visualize high dimensional single cell data in two dimensional (tSNE-x tSNE-y parameters) graphs to qualitatively assess cell population diversity^52,53^. Clustering of cells is based on their similarity in composition and expressed markers and clusters are positionally adjacent on the two-dimensional scatter plots of the t-SNE graphs. t-SNE analyses were performed using standardized parameters across all samples: perplexity = 30, iterations = 1000, and learning rate (Eta) = 200. A third dimension may be applied to visualize the intensity of each marker expression by using a colour-based heatmap (Fig. 2A). All analyses were performed using the FlowJo 10 bioinformatic software.

### Statistical analysis

Statistical analyses were performed according to the two-tailed Student’s *t* test for dual comparison (treated versus control samples). Data are expressed as mean values ± standard error of measurement (SEM) and significant *P*-values.

All methods were carried out in accordance with relevant guidelines and regulations.

## Results

### Cell population subsets identified by flow cytometry and in vitro differentiation trajectory towards the myeloid lineage of bone marrow cells from patients with AML and MDS

In order to identify population subsets based on the expression of the selected markers, all bone marrow samples, non-treated, Imiquimod- and R848-treated from MDS and AML patients, were grouped into a single concatenated file. First, following manual gating, after excluding lymphocytes, mononuclear phagocytes and selecting CD45^+^ leucocytes, seven cell population clusters were identified (Fig. 1). t-SNE analysis of the seven subsets is represented in Fig. 2A, along with heat-maps of the marker expression levels. The most immature cells are clustered in populations A and B (CD34^+^ CD11b^–^); cells showing an intermediate differentiation degree are clustered in populations C and D (CD34^–^ CD11b^–^); more mature cells are grouped in clusters E, F and G (Figs. 1B and 2). In addition, subsets B and C differ from clusters A and D, respectively in the expression of CD117 (a marker of progenitor cells) and CD13 (a marker of myelogranulocytes) which are expressed in B and C cell subsets (Figs. 1B and 2A). Cluster G shows high expression of CD117 and CD13 markers. Cells in cluster E are CD13^–^ and CD11b^low^, whereas cluster F are CD13^+^ and express high levels of CD11b. Both clusters E and F show the highest level of SSC (Figs. 1B and 2A), a parameter that measures cell complexity and increases during the differentiation process and that is particularly high in granulocytes due to the presence of cytoplasmic granules.

Expression of cell markers in population A (CD34^+^, CD17^–^, CD13^–^ and CD11b^–^) and in population G (CD34^–^, CD117^high^, CD13^high^ and CD11b^+^) suggested that both cell subsets do not belong to the granulocytic series, and therefore the presence of both populations was analysed in each individual sample. Results showed that both cell subsets were mainly found in only a few patients with AML: 90% of cells in the A population were detected in four patients (40%, 26%, 17% and 7% for patients 14, 12, 17 and 10, respectively), while 79% of cells in the G populations were found in only four AML patients (33%, 23%, 16% and 7% for patients 11, 18, 9 and 13, respectively) (Fig. 2B). Consequently, as A and G subsets were only present in a few samples (4 out of 19), both populations were excluded for further analyses.

Further analyses of the expression of cell markers in the selected cell subsets (populations B-F) showed that they fit well with the previously described path of differentiation of granulocytic series towards neutrophils (Fig. 2C)^54,55^. B population corresponds to the most immature cells, showing a myeloblast phenotype; cells of C and D subsets showed an expression pattern of markers for promyelocytes and myelocytes, respectively; population E showed a pattern of marker expression corresponding to more mature cells, such as metamyelocytes, and cells of F population showed a phenotype of mature neutrophils. Therefore, all the cell subsets of the granulocytic differentiation path towards neutrophils were identified, as shown in the t-SNE analysis (Fig. 2C).

### Effect of Imiquimod and R848 on the in vitro differentiation path of AML and MDS samples

In order to determine the effect of the TLR ligands (Imiquimod and R848) on the differentiation path of AML cell samples, B-F cells subsets were analysed based on the expression of the selected markers in each treated sample from AML patients and compared to control untreated cells. AML control cells exhibited a high proportion of immature B and C cells (myeloblasts and promyelocytes) in all cases, and Imiquimod or R848 treatment did not induce cell differentiation towards more mature cell subsets of the granulocyte series, neither after 24 h nor 48 h of treatment (results not shown). Results showed wide variability among samples, and only in two cases (patients 12 and 13) out of 13 a slight differentiation was observed, although data were not statistically significant (not shown).

**Fig. 3:**
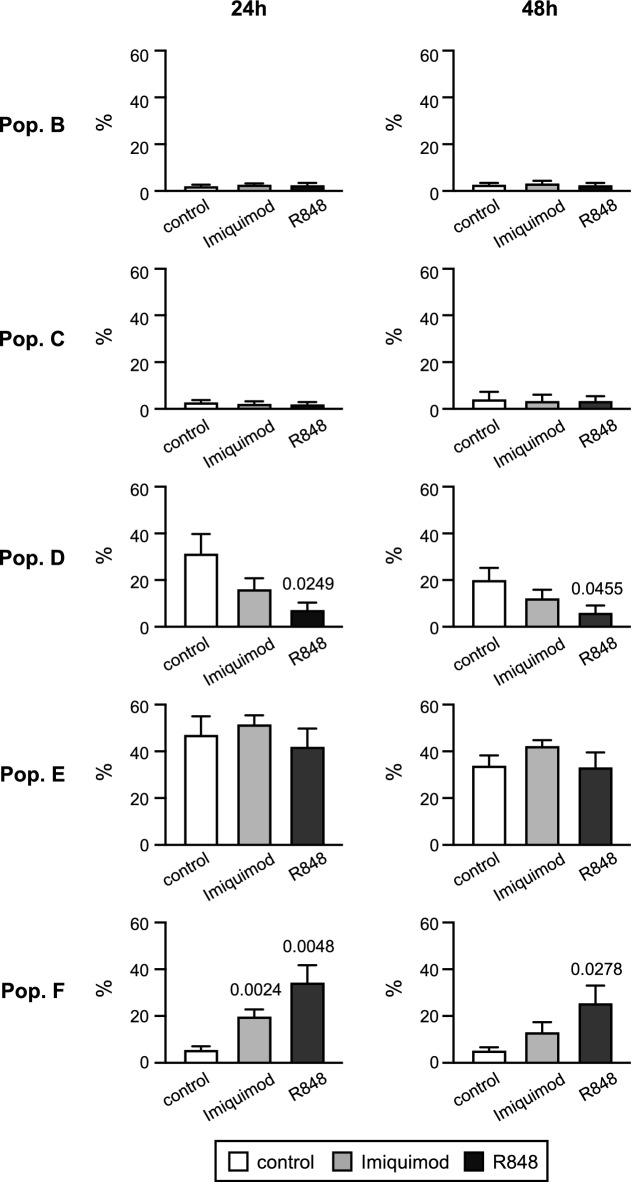
Effect of R848 and Imiquimod treatments on the cell populations in MDS samples. The percentage (mean values ± SEM) of each cell population (B-F) was determined in single MDS samples (n = 5) after 24 h and 48 h of R848 and Imiquimod treatment; untreated samples were used as controls.

When the effect of TLR ligands was analysed in MDS samples, similar modifications in the differentiation path (measured as proportion of B-F cell subsets) were observed in five (patients 1–5) out of six samples. Sample of patient 6 was excluded from the study as it showed a different response and this patient had a rare MDS type (Table 1)^50,56^. Results obtained with the five MDS samples are summarised in Fig. 3. As expected, MDS samples lack significant levels of immature cells (B and C subsets) and neither Imiquimod nor R848 caused any observable change on these cell populations. Proportion of still immature cells such as myelocytes (D cell subset) represented 31.5% and 20.2% of total cells in control untreated samples after 24 h and 48 h of culture, respectively, and these percentages decreased in R848-treated cells (7.4% and 6.2%, at 24 h or 48 h respectively) as well as in Imiquimod-treated cells (16.3% at 24 h and 12.3% at 48 h). Despite treatment with both ligands showed a similar effect, only R848 caused a statistically significant decrease. Population E (metamyelocytes) was the most abundant in control untreated MDS samples (47.2% and 34.0% of total cells at 24 h and 48 h of cell culture, respectively). Treatment with either Imiquimod or R848 did not cause significant changes in these percentages (51.6% and 42.4% at 24 h and 48 h, respectively for Imiquimod and 42.1% and 33.2%, at 24 h and 48 h, respectively for R848). The levels of F cell population (neutrophils) in control untreated samples were clearly lower than immature precursors (D and E subsets) as expected for MDS samples (5.7% and 5.4% at 24 h and 48 h of culture). Interestingly, treatment for 24 h with TLR ligands caused a statistically significant increase in the proportion of mature cells (34.5% in R848-treated samples, and 20.0% in Imiquimod-treated samples). Similar results were obtained following 48 h of treatment (25.6% for R848 and 13.2% for Imiquimod), although only significant differences were found for R848 treatment. These results strongly suggest that both TLR ligands, particularly R848, are able to induce differentiation of MDS cells towards mature neutrophils, since the decrease in myelocytes (population D) is accompanied by an increase in mature neutrophils (population F), whereas the intermediate metamyelocytes (population E) remain roughly constant, as differentiation towards neutrophils is counterbalanced by differentiation from myelocytes.

**Fig. 4:**
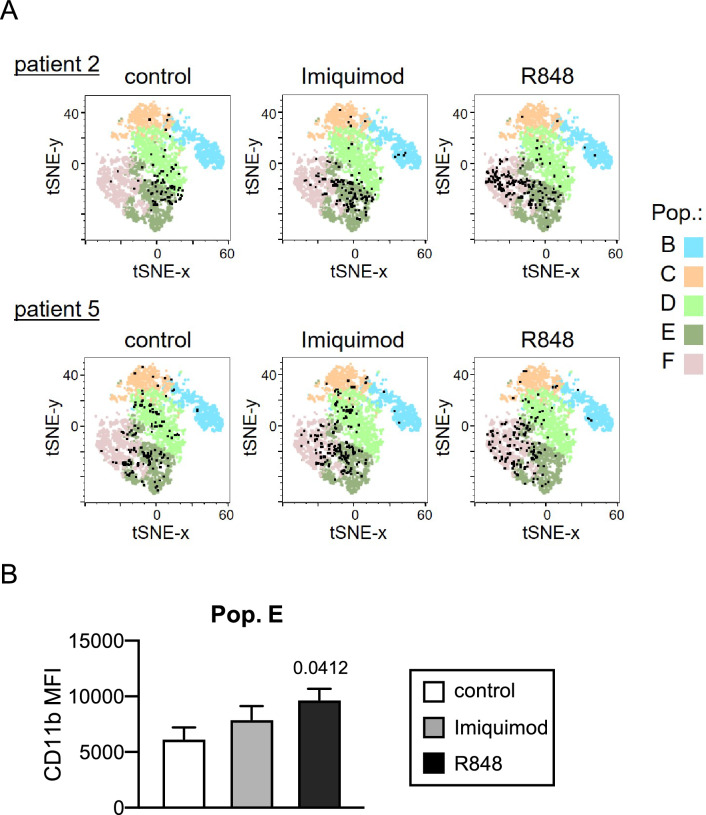
Effect of the R848 and Imiquimod treatments on cell differentiation in bone marrow cells from MDS patient samples. (**A**) Location of single cells (black dots) from patient 2 and patient 5 is shown on the t-SNE generated graph after 24 h and 48 h of treatment with R848 and Imiquimod treatment; untreated samples were used as controls. (**B**) Expression of CD11b marker in population E of bone marrow samples from MDS patients after 24 h of R848 and Imiquimod treatment; untreated samples were used as control. Mean intensity values ± SEM are shown.

The effect of both TLR ligands on the differentiation of MDS samples was further assessed by showing the distribution of cells from individual samples (from patient 2 and patient 5 as representative samples) on the differentiation path obtained in the t-SNE analysis (Fig. 4A). In both cases, a tendency is observed for cells to move from population D to population F after 24h treatment with R848 or with Imiquimod.

Although no significant changes were observed in the percentage of cells in population E after treatment with both TLR ligands (as shown in Fig. 3), and this cell subset showed low levels of CD11b expression (as shown in Fig. 2A and C), expression levels of CD11b were quantified in these cells (population E). R848 caused an increase in CD11b expression (Fig. 4B), which indicates an enhanced differentiation of these cells, supporting that R848 promotes differentiation of MDS cells from D population to mature neutrophils (F population).

To find out whether differential TLR expression could underlie the distinct responses to TLR7/8 ligands observed between MDS and AML samples, we obtained transcriptomic data from the BloodSpot database (http://www.bloodspot.eu). As shown in Supplementary Figure S, TLR7 expression is relatively constant across healthy bone marrow, MDS, and AML subtypes. In contrast, TLR8 expression exhibits more variability. Notably, MDS samples show high TLR8 expression levels comparable to healthy bone marrow, whereas AML samples generally show reduced TLR8 expression with substantial variability between AML subtypes. These data suggest that elevated TLR8 expression in MDS could contribute to the observed sensitivity to R848-induced differentiation, while reduced TLR8 expression in AML may limit responsiveness.

### In vitro effect of Imiquimod and R848 on cell viability and apoptosis in AML and MDS samples

**Fig. 5:**
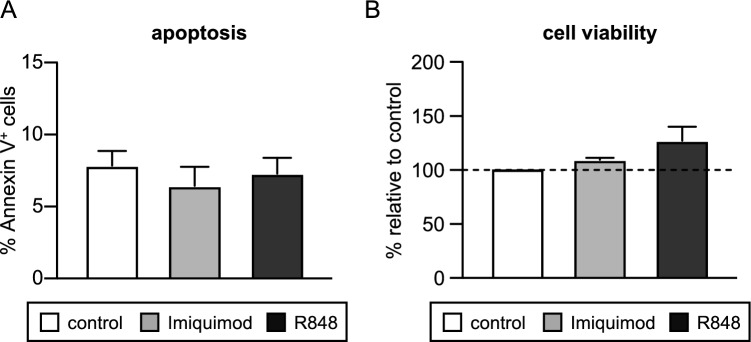
Effect of R848 and Imiquimod treatment on apoptosis and cell viability of bone marrow cells from MDS patients. (**A**) Percentage of Annexin V positive cells, determined by flow cytometry analysis, in MDS samples treated for 24 h with R848 and Imiquimod and untreated control samples; mean values ± SD are shown. (**B**) Percentage of viable cells (measured by a colorimetric assay) in MDS samples treated for 24 h with R848 and Imiquimod and untreated control samples; mean values ± SEM are shown relative to control.

As indicated in the Background section, R848 and Imiquimod are able to induce apoptosis in some cancer cells, and Imiquimod displays anti-proliferative and pro-apoptotic effects in an AML cell line (HL-60)^35–37,39,41,42^. Therefore, we determined the effect of both TLR ligands on apoptosis and cell viability in AML and MDS bone marrow samples. Minor changes on apoptotic levels and cell viability were observed when AML samples were treated with ligands, however changes were not consistent among samples (data not shown) and therefore no clear conclusions were obtained. When MDS cells were analysed, no statistically significant differences were found between control and treated samples in apoptosis (Fig. 5A) and cell viability (Fig. 5B), although a tendency to increase cell viability was observed after treatment with R848. These results indicate that R848 and Imiquimod do not show a toxic effect on MDS cells, since they do not induce apoptosis or decrease cell viability.

## Discussion

TLR expression on haematopoietic stem and progenitor cells (HSPC) and their role in inducing cell differentiation laid the foundations for developing a new strategy to deal with cancer treatment including haematological neoplasms, such as AML and MDS^25,31–33,42^. In this work we have shown that TLR7 and TLR8 agonists are able to induce in vitro differentiation of bone marrow cells from MDS patients, although no effect was found in samples from AML patients.

We used a combined approach of conventional flow cytometry (manual gating) and t-SNE analysis. First, we characterized the cell populations of the myeloid series present in bone marrow samples from AML and MDS patients by flow cytometry. Based on the expression of cell markers five major subsets of cell populations of the granulocytic differentiation path towards mature neutrophils were found, according to previous observations^54,55^. Interestingly, the dot graphs generated by the t-SNE analysis confirmed the results obtained following the manual gating, as all subpopulations were grouped in five independent homogenous cell clusters. Therefore, the differentiation path towards neutrophils was also clearly shown by the t-SNE analysis. Besides, these results confirm the high correlation between conventional and t-SNE gating in bone marrow samples, previously described for human blood cell subsets^53^.

Percentages of each population subsets from AML and MDS samples were in accordance with the WHO diagnostic criteria and the international consensus classification of myelodysplastic disorders^49–51^. MDS samples contained very low levels of immature progenitor cells (blasts and promyelocytes) and higher levels of more differentiated intermediate cells (myelocytes and metamyelocytes) including a minor population of mature neutrophils. This pattern of cell subsets fits with the blockade of the differentiation path at the level of myelocytes previously described in MDS. By contrast, AML samples contained high levels of immature cells as expected (blasts and promyelocytes).

Our results show that treatment with R848 (a TLR7/TLR8 ligand) or with Imiquimod (a TLR7 agonist) does not induce significant changes in cell differentiation in AML samples, although the observed effects varied among samples. It should be noted that AML samples were from patients aged from 40 to 81 years, and the possible influence of immunosenescence in these results remains to be determined, although it cannot be ruled out, as immunosenescence might affect distribution of leukaemic cell subsets as well as their response to TLR ligands and/or expression of TLRs^57–59^. A previous report showed that R848, but not Imiquimod, induces differentiation of primary bone marrow cultures from AML patients^33^. This discrepancy can be due to differences in methodology and/or the heterogeneity of in vitro responses in AML samples. In fact, R848 has been shown to induce differentiation specifically in *DNMT3A*-mutant AML cells^34^. Interestingly, two samples (patients 12 and 13) showed a modest trend towards differentiation upon R848 treatment, suggesting that these particular cases may harbour genetic features that confer sensitivity to TLR8 activation. Although we did not perform mutational profiling of our AML cohort, it is plausible that these two patients carry DNMT3A or other mutations, whereas the non-responsive samples likely do not. However, our results show that both R848 and, to a lesser extent, Imiquimod are able to induce differentiation of bone marrow cells from myelocytes to mature neutrophils in most MDS samples, despite MDS include heterogeneous disorders^8,51^. A potential limitation of this study is the relatively small sample size of MDS patients (n = 6). However, despite the known clinical and biological heterogeneity of MDS, we observed a consistent differentiation response to R848 in five out of six samples. This high degree of concordance supports the robustness of our results and suggests that TLR7/8 activation may elicit a reproducible biological effect in a majority of MDS cases. Nevertheless, further studies involving larger cohorts will be necessary to explore potential correlations with specific MDS subtypes or genetic features. The differential response to R848 between MDS and AML samples may be at least partially explained by differences in TLR8 expression. Publicly available transcriptomic data revealed that TLR8, but not TLR7, is highly expressed in MDS samples at levels comparable to healthy bone marrow, while expression in AML is generally reduced and heterogeneous. This could also explain that the combined activation of both TLR7 and TLR8 (in response to R848) causes a major effect on cell differentiation than activation of TLR7 alone (in response to Imiquimod). These findings are consistent with our in vitro data and support the hypothesis that differentiation induced by R848 could depend in part on TLR8 expression levels. This suggests that TLR8 expression could potentially serve as a predictive biomarker of response to TLR7/8 agonists in myeloid malignancies.

Previous studies have shown that TLR ligands can regulate haematopoiesis through both direct, receptor-mediated mechanisms and indirect pathways involving the production of inflammatory cytokines^60–62^. Although cytokine levels were not measured in the present study, it has been reported that human CD34⁺ haematopoietic progenitors secrete IL-6 and GM-CSF upon stimulation with R848, which may contribute to myeloid differentiation in a paracrine or autocrine manner^24^. Further research will be required to characterise the cytokine milieu as well as the downstream signalling pathways and gene expression programmes activated by TLR7/8 stimulation in MDS cells. Such mechanistic investigations could help to clarify whether the observed differentiation reflects a physiological enhancement of myelopoiesis or a response resembling direct pathogen sensing.

Previous reports indicated that R848 and Imiquimod can induce apoptosis in some types of cancer cells, and that Imiquimod has an anti-proliferative and pro-apoptotic effect in AML cell lines^32,35–38^. Our results showed absence of toxic effects of both ligands on primary cultures of bone marrow cells from MDS patients, as both apoptosis and cell viability were not altered. As for the differentiation assays, the effect of both ligands on apoptosis and cell viability in primary cultures from AML patients was unclear, mainly due to the heterogeneity of results among samples, as mentioned above. Therefore, the main effect of R848 and Imiquimod on MDS samples is the induction of differentiation towards neutrophils.

From these results we can hypothesise that treatment with TLR7/8 ligands might revert the blockade of myeloid differentiation in MDS patients and increase the amount of neutrophils. This could benefit patient’s survival, (i) by diminishing the risk of infection, since neutropenia is a major cause of serious/lethal infections in these patients^9,14^, and (ii) by reducing the probability of progression from MDS to AML, since mature neutrophils have lost the proliferative properties of the precursor immature cells, preventing accumulations of further mutations^2,5^. Thus, a combined therapy with classical chemotherapy and TLR ligands could improve the efficacy of treatment and patient prognosis. Besides, our results also support the notion that new strategies of TLR-based treatments for MDS may act directly against cancer. In fact, it is well known that treatment of promyelocytic leukaemia with ATRA, a molecule that induces differentiation of promyelocytes and reverts the blockade of myeloid differentiation observed in these patients, results in high survival rates^22^. Similarly, the induction of the differentiation of myelocytes in response to R848 and Imiquimod, to a lesser extent, could represent also a potential successful treatment of most MDS patients. Further studies are needed, including in vivo testing of R848/Imiquimod treatments in animal models of MDS, and to determine their systemic effects, as TLRs are present in a wide variety of cell types with multiple immune and non-immune functions. In fact, in addition to direct effects on differentiation of MDS cells, in vivo TLR7/8-mediated activation of other immune cells could induce cell-mediated immune responses to tumour cells, also contributing to enhance their therapeutic effect^43,44^.

Finally, it should be stressed the importance of t-SNE analysis in defining in a higher detail all cell clusters present in AML and MDS bone marrow samples. It has been shown that bone marrow stem cell architecture can drive progression of MDS and may predict response to second-line treatments^7^, and more recently, it has been described the presence of leukaemia-related cell clusters which may be very helpful in the context of minimal disease detection and antigen-targeted therapies^63^.

## Conclusions

R848 and, to a lesser extent, Imiquimod induce in vitro differentiation of primary bone marrow cell cultures to mature neutrophils in most MDS samples (but not in AML samples), without significantly affecting apoptosis and cell viability. Therefore TLR7/8-mediated signalling plays a role in inducing differentiation of intermediate cell populations of the myelogranulocyte differentiation series. Consequently, treatments based on TLR7/8 ligands might revert the blockade of myeloid differentiation in MDS patients and increase the number of neutrophils, and therefore have benefits for the patient’s survival and probably improve the efficacy of chemotherapy treatment and patient prognosis.

## Supplementary Information

Supplementary Information 1.

Supplementary Information 2.

## Data Availability

The data supporting the conclusions of this paper are included within the manuscript. Raw data of Figs. 2B, 3, 4B and 5 are provided as Additional file 1.

## Abbreviations

AML: Acute myeloid leukemia
MDS: Myelodysplastic syndromes
HSPC: Hematopoietic stem and progenitor cells
TLR: Toll-like receptors
BM: Bone marrow
t-SNE: T-distributed stochastic neighbour embedding
ATRA: All-trans retinoic acid
