# Supplementary material for: TLR7/8 ligands R848 and imiquimod induce differentiation of bone marrow cells from patients with myelodysplastic syndrome towards mature neutrophils
Source: Sci Rep. 2025 Aug 26;15:31496. doi: 10.1038/s41598-025-15859-z (PMC12381240; doi:10.1038/s41598-025-15859-z)
Supplement: Supplementary file 2 — Supplementary Information 2. [file 41598_2025_15859_MOESM2_ESM.pdf]

Figure S

A

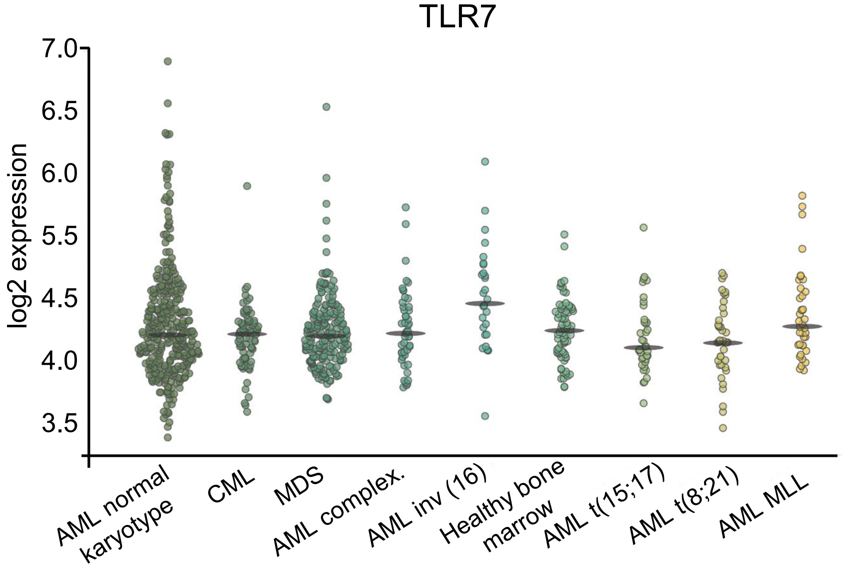

B

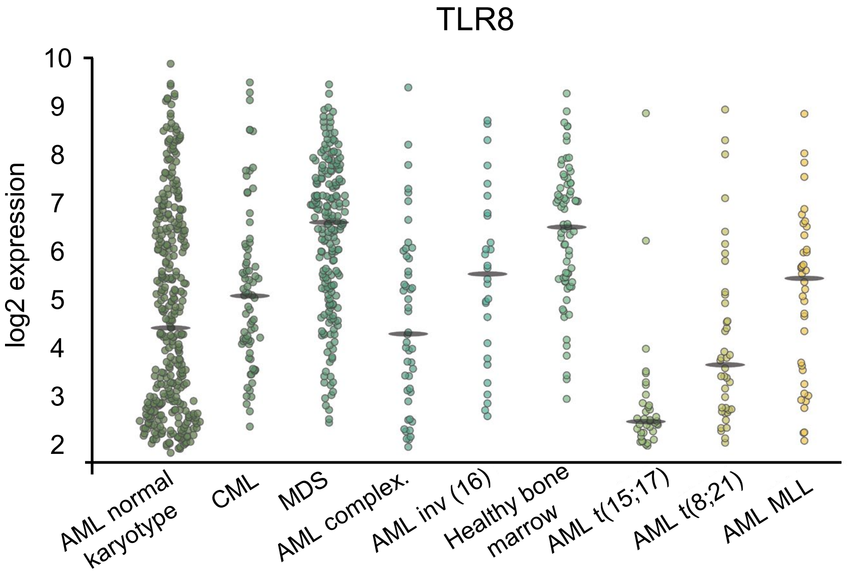

Figure S. Expression levels of TLR7 and TLR8 in healthy bone marrow, MDS, and AML samples. Gene expression data were obtained from the public transcriptomic database www.bloodspot.eu, specifically from the Microarray Innovations in Leukemia (MILE) study (GEO accession: GSE13159) [1]. Data are shown as individual samples (circles), with horizontal bars representing the median of each group. Selected samples: AML normal karyotype, AML with normal karyotype plus other abnormalities; CML, Chronic Myeloid Leukaemia; MDS, Myelodysplastic Syndromes; AML complex, AML complex aberrant karyotype; AML inv(16), AML with inv(16)/t(16;16); Healthy bone marrow, non-leukemia and healthy bone marrow; AML t(15;17), AML with t(15;17); AML t(8;21), AML with t(8;21) and AML MLL, AML with t(11q23)/MLL.

1. Bagger FO, Sasivarevic D, Sohi SH, Laursen LG, Pundhir S, Sønderby CK, Winther O, Rapin N, Porse BT. BloodSpot: a database of gene expression profiles and transcriptional programs for healthy and malignant haematopoiesis. Nucleic Acids Res 2016, 4;44(D1):D917-24.
